# Changes in heart rate variability induced by E-sports activities

**DOI:** 10.3389/fphys.2025.1557579

**Published:** 2025-04-22

**Authors:** Ti Wu, Po-Yao Lee, Jie-An Tu, Hsin-Huan Wang, Hsueh-Chin Chao, Chia-Hsiang Chen, Jui-Hung Tu

**Affiliations:** ^1^ Graduate Institute of Sports Science, National Taiwan Sport University, Taoyuan, Taiwan; ^2^ Department of Physical Education, National Pingtung University, Pingtung, Taiwan; ^3^ Office of Physical Education, National Pingtung University of Science and Technology, Pingtung, Taiwan; ^4^ Department of Tourism and Leisure Management, Yu Da University of Science and Technology, Miaoli, Taiwan; ^5^ Physical Education Office, National Kaohsiung University of Science and Technology, Kaohsiung, Taiwan

**Keywords:** autonomic nervous system, fatigue monitoring, physiological responses to gaming, gaming, HRV (heart rate variability)

## Abstract

**Introduction:**

In recent years, E-sports has emerged as a global competitive sports discipline. However, research in the field of exercise science regarding this burgeoning area remains relatively limited. Within this context, an in-depth exploration of E-sports from an exercise science perspective not only aids in optimizing players’ training and performance but also contributes important theoretical and practical value to the advancement of exercise science. This study investigated the physiological changes in E-sports athletes by measuring Heart Rate Variability (HRV).

**Methods:**

A total of 40 male college students (mean age 21.2 ± 2.4 years, mean height 171.8 ± 7.9 cm, and mean weight 70.2 ± 11.5 kg) were recruited. Heart rate data were collected before, during, and after gaming using SCOSCHE heart rate armbands. HRV Analysis 1.1 software was employed to convert heart rate changes into HRV parameters. First, a normality test was conducted to determine the data distribution. Since the data did not meet the assumption of normality, the Friedman test was used to compare pre-, during-, and post-gaming heart rate data. Post hoc comparisons were performed using the Wilcoxon signed-rank test with Bonferroni correction. The significance level was set at α = 0.05.

**Results:**

The results showed that E-sports gaming significantly affected autonomic nervous system activity. Specifically, pre-game mean heart rate (Mean HR) and low-frequency (LF) power were significantly lower than those measured during and after gaming (*p* < 0.05), while pre-game mean RR interval (Mean RR) was significantly longer (*p* < 0.05). Furthermore, pre-game HRV indices [e.g., Standard Deviation of NN intervals (SDNN), Root Mean Square of Successive Differences (RMSSD), Number of NN intervals differing by more than 50 ms (NN50), Percentage of NN50 (pNN50)] and high-frequency (HF) power and LF/HF Ratio were significantly higher than those recorded during and after gaming (*p* < 0.05).

**Conclusion:**

E-sports gaming imposes stress and fatigue on HRV-related parameters, heightening sympathetic activity and suppressing parasympathetic function. Although certain indicators exhibited a trend toward recovery during the post-gaming rest period, complete recovery appears to require a longer duration. These findings confirm the utility of HRV as an essential tool for monitoring athletes’ physiological status and underscore the need for effective recovery strategies to mitigate the long-term impact of gaming-related stress.

## Introduction

Despite the lack of a universally accepted definition, electronic sports (E-sports) are traditionally described as a form of “sport” and a competitive activity, primarily involving online multiplayer virtual battles conducted by players through electronic devices (computers or mobile phones) (Sjöblom and Hamari, 2017). Although E-sports games and competitions have been held for some time, the prevalence of COVID-19 in recent years has made online gaming an important form of entertainment for people. Many also watch E-sports competitions through online streaming platforms (e.g., YouTube, Twitch, or Smashcast), leading E-sports s to rapidly emerge as a new form of media. In 2021, there were more than 2.6 million mobile game players worldwide, with half of them located in Asia (Lam et al., 2022). In mainland China, 560 million people regularly play computer or mobile games, accounting for 70% of the country’s online population, driving E-sports industry revenues to USD 11.1 billion in 2016 (Chung et al., 2019). Research indicates that the global es E-sports industry generated USD 152 billion in revenue in 2019, and it is predicted to grow to USD 257 billion by 2025 (Truong et al., 2020). The recently concluded 2024 League of Legends World Championship reached an all-time high viewership, with global concurrent viewers approaching seven million (Iyer, 2024).

Although the E-sports market has grown, there has been limited research on its physiological demands. E-sports athletes share many similarities with traditional athletes, such as undergoing training, participating in competitions, and adhering to rules set by teams or associations. They also face similar psychological and physiological challenges to traditional athletes—such as pre-competition stress, training or competition-related injuries, and relatively short career spans. Hence, E-sports and traditional sports have many parallels (Nagorsky and Wiemeyer, 2020). However, health promotion is mainly associated with traditional sports; systematic endurance training helps improve cardiovascular health, and muscular activity in endurance or strength training has been proven effective in preventing many lifestyle diseases (Chen et al., 2016). Whether E-sports can also offer these benefits remains uncertain. Currently, our understanding of E-sports athletes in exercise science is still limited.

## Physiological changes and fatigue monitoring in E-sports athletes

E-sports athletes often spend 3–10 h per day training or competing to develop excellent hand–eye coordination and reaction times, and they typically do not engage large muscle groups. Prolonged static postures may increase the risk of mortality, cardiovascular diseases, and Type II diabetes (Katzmarzyk et al., 2019). Cardiovascular health is influenced by numerous factors, among which exercise is an important way to strengthen the heart (Pluim et al., 1999). Different types of exercise and training intensities affect how the heart responds to physical activity (Rasmussen et al., 2023). In recent years, Heart Rate Variability (HRV) has been used in the field of sports performance as a training indicator (Buchheit, 2014). This noninvasive marker of autonomic cardiovascular regulation is considered one of the monitoring indicators during or after exercise (Plews et al., 2013). HRV can be used to interpret the functions of the sympathetic and parasympathetic nervous systems by examining the intervals between heartbeats and heart rate frequency. Studies recording changes in the autonomic nervous system before and after exercise, particularly the regulation of the vagal (cardiac vagal) and sympathetic nerves, can shed light on how athletes’ cardiovascular functions adapt to training (Sloan et al., 2011) and help monitor exercise-induced fatigue (Buchheit et al., 2013). HRV analysis is also used to track cardiovascular health and optimize training intensity for athletes (Oliveira et al., 2013). Long-term training can increase HRV (Prinsloo et al., 2014). Exercise training promotes increased parasympathetic tension at rest and suppression of the sympathetic nervous system (Abad et al., 2014).

Traditionally, HRV measurement has relied on electrocardiograms and similar devices, which are not easily adaptable for long-term observation in daily life. In recent years, advances in wearable technology have made it possible to integrate health-related physiological data to detect, monitor, and prevent cardiovascular diseases (Rodrigues et al., 2022). In E-sports, players’ heart rate and blood pressure may increase due to catecholamine release, indicating reduced parasympathetic activity and increased sympathetic activity. During competitions, this autonomic regulation becomes particularly evident (Sousa et al., 2020). For example, when playing Fortnite, both average and peak heart rates rise under different in-game scenarios, reflecting E-sports athletes’ physiological responses to high-pressure situations (Valladão et al., 2020).

This study analyzes physiological changes and autonomic nervous system regulation before, during, and after gaming by examining fluctuations in HRV. It is hypothesized that gaming induces an increase in sympathetic nervous activity and a decrease in parasympathetic nervous activity, leading to significant alterations in HRV indices, reflecting the accumulation of physiological fatigue or stress. Furthermore, HRV parameters may not immediately return to baseline levels post-gaming, indicating a sustained impact of gaming on autonomic nervous function. The findings of this study will contribute to fatigue monitoring, training program optimization, and physiological assessment for E-sports athletes, enhancing performance and promoting health management.

## Methods

### Participants

Following approval by the Institutional Review Board of Pingtung Antai Hospital, a total of 40 male college students were recruited (mean age: 21.2 ± 2.4 years, mean height: 171.8 ± 7.9 cm, mean weight: 70.2 ± 11.5 kg). Eligibility criteria included: (1) between 18 and 25 years of age, and (2) playing E-sports for at least 6 h per week over the past year. Exclusion criteria were any upper or lower extremity neurological, muscular, skeletal, tendon, or ligament injury within the past 6 months, a history of cardiovascular disease, or current use of medications known to affect cardiac or endocrine function (e.g., blood pressure, heart rate). Prior to participation, all subjects were informed of the study’s purpose, procedures, and associated precautions, and provided written informed consent.

### Protocol

Prior to measurement, participants were instructed to abstain from caffeine, smoking, and alcohol consumption for at least 12 h. Before the experiment began, they were required to complete an informed consent form and provide their exercise and esports history, including weekly frequency of exercise sessions, hours of exercise per week, and hours of esports play per week. In addition, each participant’s height and weight were recorded.

Participants then sat resting for 5 min to ensure a stable resting state. Subsequently, a SCOSCHE heart rate armband (SCOSCHE, California, USA) was connected via Wi-Fi to a WIMU PRO tracking system to collect 10 min of resting heart rate data (denoted as Pre-test). During this period, participants were instructed not to use their phones, engage in conversation, or fall asleep. Following the resting heart rate assessment, they proceeded to play the game. The experimental participants played League of Legends, a Multiplayer Online Battle Arena (MOBA) game. As soon as the game match began, heart rate data were collected throughout the game, totaling two games (labeled G1 and G2). The duration of two games was approximately one and a half hours. After the completion of game G2, participants rested in a seated position for 30 min, again refraining from phone use, conversation, or sleeping. At the end of this 30-min interval, another 10 min of resting heart rate data were collected (labeled Post-test).

### Statistical analysis

Heart rate data (Mean HR and Mean RR) from the participants were collected using the WIMU PRO tracking system and a heart rate armband. Through the SPRO analysis software, the data were displayed in real time on a computer for immediate observation of any irregularities. Subsequently, the HRV Analysis 1.1 software was employed to convert the Mean RR values into additional indices, including the Standard Deviation of NN intervals (SDNN), Root Mean Square of the Successive Differences (RMSSD), the number of pairs of successive NN (NN50), the proportion of NN50 (pNN50 [%]), Low-Frequency power (LF), High-Frequency power (HF), and the LF/HF Ratio (LF/HF).

Statistical analyses were performed using IBM SPSS 22.0 (SPSS Inc., Chicago, United States). First, a normality test was conducted to determine the data distribution. If the normality assumption was violated (*p* < 0.05), the Friedman test was used to compare differences among Pre-test, G1, G2, and Post-test values. Post hoc comparisons were conducted using the Wilcoxon signed-rank test with Bonferroni correction. The significance level was set at α = 0.05.

## Results

In this study, normality tests were performed to determine the distribution of the data. Since the assumption of normality was violated (*p* < 0.05), the Friedman test was employed to compare the differences among the pre-test, G1, G2, and post-test values. Post-hoc comparisons were then conducted using the Bonferroni-adjusted Wilcoxon signed-rank test.

As shown in Table 1, the comparison of pre- and post-esports gaming HRV revealed that the stress and fatigue induced by gaming led to notable changes in several parameters. Mean HR prior to gaming was significantly lower than that during G1, G2, and after gaming (*p* < 0.05). Mean RR before gaming was significantly longer than that during G1, G2, and after gaming (*p* < 0.05). Following a 30-min rest period, the Mean RR measured post-gaming was significantly longer than during G1 and G2.

**TABLE 1:** Heart rate variability changes before, during, and after playing E-sports games.

	Pre-test	G1	G2	Post-test	*p*
Mean HR	74.6 ± 10.7	81.35 ± 12.78^#a^	80.51 ± 11.94^-b^	78.47 ± 10.99^∗^	0.000
Mean RR	824.6 ± 113.61	744.3 ± 104.9^#a^	762 ± 109.38^-b^	792.4 ± 109.76^∗^	0.000
SDNN	57.81 ± 18.97	38.71 ± 13.28^#a^	43.21 ± 11.66^-b^	49.9 ± 17.1^∗^	0.000
RMSSD	19.14 ± 10.43	11.76 ± 4.74^#a^	12.66 ± 5.65^-b^	16.49 ± 8.39^∗^	0.000
NN50	1.9 ± 2.33	1.1 ± 1.46^#^	1.0 ± 1.63^-^	1.0 ± 1.78^∗^	0.000
pNN50 (%)	0.19 ± 0.27	0.14 ± 0.19^#^	0.12 ± 0.15^-^	0.14 ± 0.24^∗^	0.000
LF	309.09 ± 96.33	352.19 ± 71.79^#^	394.59 ± 148.79^-^	364.86 ± 83.23^∗^	0.000
HF	22.52 ± 3.63	15.59 ± 3.52^#^	15.77 ± 3.43^-^	20.90 ± 3.13^∗^	0.000
LF/HF	14.25 ± 5.59	24.05 ± 8.88^#^	25.31 ± 9.39^-^	17.74 ± 4.54^∗^	0.000

Note: Values are presented as the mean ± SD.

^∗^Indicates significant difference were observed between pre-gaming and post-gaming conditions.

^#^Indicates significant difference were observed between pre-gaming and G1 conditions.

^−^Indicates significant difference were observed between pre-gaming and G2 conditions.

^a^Indicates significant difference were observed between post-gaming and G1 conditions.

^b^Indicates significant difference were observed between post-gaming and G2 conditions.

(*p* < 0.05).

Regarding SDNN, pre-gaming SDNN was significantly higher than that during G1, G2, and after gaming (*p* < 0.05), and remained significantly higher than G1 and G2 after the 30-min rest. Similarly, RMSSD was significantly higher pre-gaming compared to G1, G2, and post-gaming (*p* < 0.05). After resting, the post-gaming RMSSD value was still higher than the values for G1 and G2. For NN50, the pre-gaming value was significantly higher than during G1, G2, and post-gaming (*p* < 0.05). In pNN50, the pre-gaming value was likewise significantly higher than that of G1, G2, and post-gaming (*p* < 0.05).

Regarding frequency domain measures, the pre-gaming LF value was significantly lower than during G1, G2, and post-gaming (*p* < 0.05). Conversely, the pre-gaming HF was significantly higher than during G1, G2, and post-gaming (*p* < 0.05).

## Discussion

### Autonomic responses during gaming

This study examined changes in heart rate variability (HRV) before, during, and after gameplay. The research hypothesis posited that sympathetic activity would increase while parasympathetic activity would decrease during gameplay. The results confirmed this hypothesis, as indicated by a significant increase in heart rate (HR) and a shortened RR interval, reflecting enhanced sympathetic activation and suppressed parasympathetic function (Ketelhut and Nigg, 2024). Additionally, a significant increase in low-frequency (LF) power and a decrease in high-frequency (HF) power were observed, consistent with previous literature (Thayer and Lane, 2000; Usui and Nishida, 2017). This suggests that in high-pressure competitive gaming environments, sympathetic activity dominates autonomic regulation, while parasympathetic modulation is inhibited (Watanabe et al., 2021; Long et al., 2023). Furthermore, substantial reductions in HRV indices such as the standard deviation of NN intervals (SDNN) and the root mean square of successive RR interval differences (RMSSD) further indicate parasympathetic suppression (Billman, 2011).

However, these changes primarily occurred during the transition from pre-game to during-game, while the transition from during-game to post-game exhibited a different autonomic modulation pattern. Specifically, although the LF/HF ratio remained elevated post-game, partial recovery in HRV indices such as SDNN and RMSSD was observed, suggesting a gradual restoration of parasympathetic activity (Billman, 2011; Kim et al., 2021). These findings partially support our hypothesis but also highlight that post-game recovery patterns differ from those typically observed following physical exercise.

### Definition of game intensity

Notably, while the present study observed an increase in heart rate (HR) during gameplay, the average HR remained at only 80 bpm. In exercise physiology, HR or rating of perceived exertion (RPE) is typically used to assess exercise intensity, and an HR of 80 bpm is insufficient to be classified as high-intensity exercise. This indicates that the definition of high-intensity gaming differs fundamentally from that of high-intensity exercise. High-intensity gaming is more closely associated with psychological stress and cognitive load, rather than a purely cardiovascular demand. Therefore, future research should further distinguish the autonomic regulatory mechanisms underlying these two conditions (Drachen et al., 2010; Smerdov et al., 2020; Krarup and Krarup, 2020).

### Post-game recovery patterns

Following gameplay, HRV indices such as SDNN and RMSSD showed partial recovery; however, the LF/HF ratio remained elevated, indicating persistent sympathetic activation (Chin et al., 2024; Yeo et al., 2018). This pattern differs from post-exercise physiological recovery, where parasympathetic activity typically rebounds more rapidly, whereas in high-pressure gaming environments, autonomic recovery appears to be slower (Pedraza-Ramirez et al., 2020; Leis and Lautenbach, 2020). Research suggests that this delayed recovery may be attributed to factors such as high-pressure challenges, cognitive load, and prolonged attentional demands during gameplay (Forcier et al., 2006; Milatz et al., 2015).

Furthermore, the findings of this study align with those of Yeo et al. (2018) and Zimmer et al (2022), which suggest that higher psychological stress and increased game challenge levels may prolong HRV recovery time. Similarly, Nakamura et al. (2016) reported that high-pressure gaming environments result in more prolonged HRV suppression and slower recovery. Future research should explore potential recovery strategies, such as breathing exercises or light physical activity, to facilitate post-game autonomic balance.

## Conclusion and recommendations

This study compared HRV before, during, and after esports gameplay, revealing a significant impact on the autonomic nervous system. The findings indicate a substantial increase in sympathetic activity during gameplay, as evidenced by a marked rise in Mean HR and a decrease in Mean RR, alongside notable reductions in HRV indicators such as SDNN and RMSSD, suggesting suppressed parasympathetic function. Moreover, the increase in LF and the decrease in HF further confirm a sympathetic-dominant regulatory pattern triggered by gaming. During the post-game recovery phase, HRV indicators gradually rebounded, although the rate of recovery remained relatively slow. In particular, the persistently elevated LF/HF ratio signifies continued sympathetic activation, a phenomenon especially pronounced under high-pressure and high-intensity gaming scenarios.

Based on these results, players are advised to schedule gaming sessions and frequency judiciously to avoid excessive autonomic load stemming from prolonged high-intensity play. Furthermore, post-game restorative activities—such as meditation, deep breathing, or light exercise—may facilitate the reactivation of parasympathetic function. For professional competitors or players frequently exposed to intense gaming, targeted stress management and recovery strategies could help mitigate adverse effects on both physical and mental health.

To improve the accuracy of future experiments, controlled breathing frequency should be incorporated to ensure consistency in HRV measurements, as variations in breathing rate can influence autonomic regulation. Additionally, breath training interventions could be explored as a potential method to stabilize HRV responses, reducing excessive fluctuations caused by prolonged gaming. Future research might investigate how different game types, player characteristics (e.g., gender, age), and long-term gaming participation influence autonomic function, thus providing a scientific foundation for healthier gaming practices and informed game design.

## Data Availability

The raw data supporting the conclusions of this article will be made available by the authors, without undue reservation.
